# Success in animal skin fashion in African countries or the boom of the wet market

**DOI:** 10.14202/vetworld.2022.1328-1332

**Published:** 2022-05-26

**Authors:** Andile Ephraim Mkhonza, Keitiretse Molefe, Odirile Thato Lebogang Ramafoko

**Affiliations:** Department of Animal Health, North-West University, Mafikeng Campus, Private Bag X2046, Mmabatho 2735, South Africa

**Keywords:** animal welfare, clothing, disease reservoirs, poverty

## Abstract

The world and the way things are done have changed, from selling clothing in brick-and-mortar stores to online shopping through social media platforms. Population growth has significantly contributed to an increased clothing demand, which, in turn, has increased the demand for animal skin. Traditional markets, also known as wet markets, are considered as major zoonotic disease reservoirs due to human and animal contact. Some groups and individuals continue to believe in traditional medicine and clothing that is made from animal skin, and such beliefs are more accessible with the presence of wet markets. Hence, animal poaching and trafficking have increased to meet the high demands, primarily in the Western world. Poverty is a well-known motivation to commit a crime. Conservationists should not only look at the animal regulation site to propose a solution to animal poaching and trafficking but should also consider communal poverty. Thus, this review aimed to highlight the role of wet market and animal skin fashion on animal welfare and human health.

## Introduction

Since the dawn of humanity, animal hides have been a common clothing material used by many cultures in Africa [1]. Our earliest known examples of dress have used the skin of animals to cover the skin of people [2]. The skins of domesticated animals were most commonly used to make animal skin clothing [3]. As the human population increases, the demand for clothing also rapidly increases to meet human demands [4]. With the animal skin fashion advances, the cost of animal skin fashion rises; thus, the introduction of wet markets came about for low-class people to afford such. A wet market, also called a public market or a traditional market, is a marketplace for selling fresh meat, fish, produce, and other perishable goods as distinguished from “dry markets” that sell durable goods, such as fabric and electronics [5,6].

Wet markets (traditional markets) play important roles in food security and local development; however, they also have negative health implications [7]. Traditional marketing channels, particularly wet markets, dominate the retailing of vegetables, animal skins, and meat [8,9], and even the well-developed countries operate this way [10]. Due to wet markets’ cultural importance, there exists extensive tourist geared information on the Web, often on websites, such as Yelp and Trip advisor, which serve as forums to share experiences [7]. Social media also contributes to the advertising of animal skin fashion from wet markets using platforms, such as Facebook, Instagram, and Twitter. Social networking platforms do not paint a complete picture of wet markets (e.g., the total number of regional wet markets and how many yearly visitors); however, they can provide opportunities to instantaneously report and detect these elusive cases, primarily traveler-related ones, and improve the epidemiological monitoring within these settings [7].

The animal skin textile and fashion industry are undeniably significant to the economy; however, this industry often operates at the expense of environmental and social factors when considering the idea of sustainability. Hence, the goal of this study is to emphasize the impact of the wet market and animal skin fashion on animal welfare and human health. Today, using animal-derived items is not only detrimental to the animals but also to the wearer [11]. Animal products, such as animal skins, are in high demand in the fashion industry, especially in luxury fashion [12]. Furniture, art, décor, jewelry, cosmetics and perfume, food, medicines, and fashion are among the key uses for livestock and wildlife skin imports. One of the areas where fashion industries can easily get fast access to animal skin and in a larger quantity is through the wet market.

Thus, this review aimed to highlight the role of wet market and animal skin fashion on animal welfare and human health.

## Animal Skin Fashion

On average, livestock products, such as meat, milk, eggs, wool, hides, and skins, account for 28% of the agricultural gross domestic product of sub-Saharan African countries, with wide variation between countries [13]. Hides and skins are the most valuable by-product of the meat industry and are normally converted into leathers [14]. Leather is a type of ancient clothing that was once used to clothe people. People who lived during the Ice Age, 500,000 years ago, were likely the first to use animal skins and hides to protect their bodies from environmental climatic extremes [15].

The increased demand for clothing in the fashion industry has increased the global production of raw materials. In 2013, the worldwide textile fiber production was 83.8 million tons, a figure that represented an annual increase of 4.5%, building on a 5.3% increase witnessed in 2012 [16]. The growing demand for material consumption in fashion is inextricably linked to consumerism and economic growth systems based on rapid product obsolescence and ever-increasing resource throughput [4]. As the population grows, so does the demand for clothing. The fashion industry is described as a “market-driven cycle of consumer desire and demand” and “a modern mechanism for the fabrication of the self” [17,18]. Fashion’s physical products of fabric and thread are traded in pursuit of psychological needs [4].

## The Donkey Hides Fashion

The wet market has contributed to an increased demand for donkey skin. A study published in 2019 by the Kenya Agricultural and Livestock Research Organization reported that the annual number of donkeys slaughtered in Kenya was five times higher than the annual donkey population growth rate, which would lead to the complete extinction of the Kenyan donkey population by 2023 if neither rate changed [19]. In China, the donkey population decreased from 10.89 million in 1994 to 2.68 million in 2018, a drop of 75.4% [20].

The growing demand for Ejiao, gelatin produced from donkey skin that is used in Traditional Chinese Medicine and cosmetic products, is putting the global donkey population at risk [21]. Ejiao’s industrial insiders report that the price of a single hide has grown from 20 yuan in 2000 to approximately 3000 yuan lately in December 2017 [22]. Ejiao is also ascribed to anti-aging and rejuvenating effects [23]. Such high prices pique the interest of African countries in illegal trades, in which donkeys are stolen and transported miles on foot or slaughtered and skinned in the bush, which is disastrous for animal welfare [21]. The fashion industry is also generally held responsible for major social issues that cause the death of >50 million animals annually [12], which is strongly associated with the intensive use of animal fibers (e.g., skin and fur) [24].

## Wet Market-related Health Risks

Wet markets are an important part of economies and a major interface between wildlife, livestock, and humans, as they bring together live and dead animals of different species and origins, potentially facilitating interspecies transmission [25,26]. In developing cities, particularly low- and middle-income countries, such as Asia and Africa, wet markets are involved in selling live animals, commonly animal products [27]. A large number of people depend on wet markets for their fresh livestock and wild animal meat. Human interaction, including retailers and customers, live animals for sale, food products, including ready-to-eat food, and wild and peri-domestic animals, all pose significant risks for emerging infectious diseases [27].

Infectious diseases include the transmission of both zoonotic epidemics and endemic diseases. Endemic zoonotic pathogens that pose a transmission risk in markets include the avian influenza virus, *Leptospira* spp., *Brucella* spp., rickettsia, and diverse foodborne bacteria and parasites (e.g., *Trichinella* spp. and *Taenia* spp.) [28-30]. Zoonotic diseases have the potential to cause global pandemics. Large-scale zoonosis outbreaks, which resulted in large numbers of deaths, have wreaked havoc on economies, political order, and societies throughout history [31].

Guan *et al*. [32] recently established a potential zoonotic origin of severe acute respiratory syndrome coronavirus (CoV), and wet markets were a possible source of the original outbreak [33]. Wet markets have also been linked to avian influenza [7,34], and foodborne *Campylobacter*, *Salmonella*, *Giardia*, and *Escherichia* *coli* are the most common pathogens in these environments, resulting in 18 million disability-adjusted life-years annually [35]. A significant increase has been found in the frequency and biological diversity of emerging infectious diseases since the late 20^th^ century [36]. These outbreaks not only caused the death of hundreds to thousands of people but also increased the risks from comorbidity factors, such as diabetes, negatively impacted economies, and caused tensions among decision-makers [37,38].

## Environmental Impact of the Wet Markets

Wet markets have both positive and negative impacts on the environment, mostly depending on how they are characterized in terms of the One Health concept. These wet markets are most readily accessible to local consumers, often sell traditional and popular foods, and promote personal relationships between buyers and sellers [7]. They play a key role in food security and community development. However, these markets undoubtedly act as an interface for bacterial and viral exchange with a high risk of cross-species transmission to humans while providing customers with animals to consume or animal-sourced foods [27]. A significant increase was found in the frequency and biological diversity of emerging infectious diseases since the late 20^th^ century [36]. The recent history of outbreaks of CoVs and avian influenza viruses has well-illustrated that these emerging zoonotic diseases, which originate from animals in wet markets, can present threats to human health [27,33]. The effect of zoonotic infections on health and the economy is likely underrated, but the importance of wet markets on livelihoods, nutrition, and psychosocial well-being is also likely underrated [27].

## Animal Poaching and Trafficking

Poaching, or the illegal capture of animals, is one of the most serious threats to biodiversity [39]. Animal poaching and trafficking were primarily motivated by consumer demand for luxury products and, in some cases, traditional medicine. Today, interactions across species are influenced by the rise of the internet and social media that facilitate illicit trade and poaching of endangered and other species across the globe [40]. Animal poaching and trafficking may occur in a wide range of circumstances, and for a large number of reasons, it may be driven by, for example, economic motivations, culture, and tradition, complete awareness of rules and laws, restrictions on traditional access to resources, lack of engagement during rule setting, and/or large-scale criminal enterprises [41]. Terrestrial mammals are internationally traded for food and as pets, and their parts are traded for ornamental use (e.g., ivory, claws, teeth, and musk), clothing (skins and furs), and traditional medicines (e.g., tiger bones, bear gallbladders, and pangolin scales) [42].

Poaching and trafficking are not new issues in Africa; however, it has become more prevalent since the late 2000s. Significant numbers of species are lost to wildlife poaching and trafficking. In addition, in recent years, trafficking has become more organized and commercialized than ever before [43]. The continent’s burgeoning black-market trade, worth hundreds of millions of dollars, is fueling corruption in Africa’s ports, customs offices, and security forces and generating fresh revenue for rebel groups and criminal networks [44]. The market for illegal wildlife goods is global, including in the United States and Europe, but growing numbers of middle- and upper-class consumers in Asia have fed the exponential jump in prices for ivory and horn [44]. Such an increase in prices motivates poachers to exploit Africa’s wildlife animals. Hence, the probability of extinction for animal species increases.

## Conclusion

Closing wet markets to prevent poaching, trafficking, and the use of animal skin in fashion is pointless because such crimes will always find a market wherever there is hunger or poverty. More realistic approaches are beginning to emerge to tackle other international crimes, such as drug trafficking and illegal animal product trading. Thus, conservationists must stop promoting regulation and policies as the only solution to the problems that come from the wet markets since it may negatively reflect on their personal beliefs about animal welfare and exploitation.

Alternatively, the approach can include the use of educational means to change the wet market status and develop a program that will train the traders in the market in terms of health and safety. Government and stakeholders should take the forefront in the trading and control of animals or animal products. Proper public health programs need to be developed, but public consultation must be provided to balance the developmental program. International security agencies need to be brought into the development of programs to facilitate traceability. Economic and tax evasion control should be included, as well as strategies that reduce the price of illegal wildlife products and increase the opportunity costs of poaching by contributing to the eradication of rural poverty.

## Recommendations

**Figure-1:**
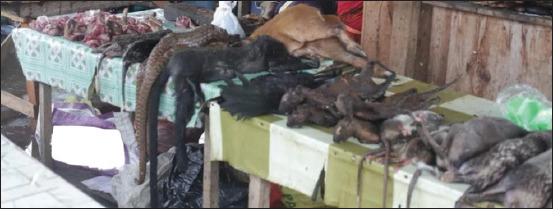
Different animals sold in a wet market [Source: https://tinyurl.com/54wt89kk].

Increased population determines an increased clothing fashion demand, which leads to an increased number of slaughtered animals, whether legally or illegally. Thus, the international trade of animals and animal products on wet markets needs to be carefully monitored and regulated. Possible traceability should be provided to both live and dead animals, including animal products that are sold on wet markets. For effective traceability, there is a need to monitor public markets that are not supervised like the one in Figure-1, which depicts the free trade of animals and animal parts. In addition, new conservation strategies must be developed that represent the powerful forces that form and control the modern world, and law enforcement of wildlife/livestock trade regulations must be considered, including the use of skin-free clothing, as well as the increased replacement of animal leather with environmentally friendly raw material. Governments and stakeholders need to invest in research studies that will allow the development of conservation and demand control.

## Authors’ Contributions

AEM: Drafted the manuscript. KM: Drafted and reviewed the manuscript. OTLR: Conceived the idea and wrote the manuscript. All authors have read and approved the final manuscript.
